# Discriminators of mouse bladder response to intravesical Bacillus Calmette-Guerin (BCG)

**DOI:** 10.1186/1471-2172-8-6

**Published:** 2007-05-16

**Authors:** Marcia R Saban, Cindy Simpson, Carole Davis, Gemma Wallis, Nicholas Knowlton, Mark Barton Frank, Michael Centola, Randle M Gallucci, Ricardo Saban

**Affiliations:** 1 College of Medicine, Department of Physiology, Oklahoma University Health Sciences Center (OUHSC), Ohlahoma City, OK 73104, USA; 2 Oklahoma Medical Research Foundation (OMRF), Microarray Research Facility, Oklahoma City, OK, 73104, USA; 3 Pharmaceutical Sciences, OUHSC, Oklahoma City, OK 73104, USA

## Abstract

**Background:**

Intravesical Bacillus Calmette-Guerin (BCG) is an effective treatment for bladder superficial carcinoma and it is being tested in interstitial cystitis patients, but its precise mechanism of action remains poorly understood. It is not clear whether BCG induces the release of a unique set of cytokines apart from its pro-inflammatory effects. Therefore, we quantified bladder inflammatory responses and alterations in urinary cytokine protein induced by intravesical BCG and compared the results to non-specific pro-inflammatory stimuli (LPS and TNF-α). We went further to determine whether BCG treatment alters cytokine gene expression in the urinary bladder.

**Methods:**

C57BL/6 female mice received four weekly instillations of BCG, LPS, or TNF-α. Morphometric analyses were conducted in bladders isolated from all groups and urine was collected for multiplex analysis of 18 cytokines. In addition, chromatin immune precipitation combined with real-time polymerase chain reaction assay (CHIP/Q-PCR) was used to test whether intravesical BCG would alter bladder cytokine gene expression.

**Results:**

Acute BCG instillation induced edema which was progressively replaced by an inflammatory infiltrate, composed primarily of neutrophils, in response to weekly administrations. Our morphological analysis suggests that these polymorphonuclear neutrophils are of prime importance for the bladder responses to BCG. Overall, the inflammation induced by BCG was higher than LPS or TNF-α treatment but the major difference observed was the unique granuloma formation in response to BCG. Among the cytokines measured, this study highlighted the importance of IL-1β, IL-2, IL-3, IL-4, IL-6, IL-10, IL-17, GM-CSF, KC, and Rantes as discriminators between generalized inflammation and BCG-specific inflammatory responses. CHIP/Q-PCR indicates that acute BCG instillation induced an up-regulation of IL-17A, IL-17B, and IL-17RA, whereas chronic BCG induced IL-17B, IL-17RA, and IL-17RB.

**Conclusion:**

To the best of our knowledge, the present work is the first to report that BCG induces an increase in the IL-17 family genes. In addition, BCG induces a unique type of persisting bladder inflammation different from TNF-α, LPS, and, most likely, other classical pro-inflammatory *stimuli*.

## Background

Intravesical Bacillus Calmette-Guerin (BCG) has been presented as a promising option for treatment of interstitial cystitis [1]. However, intravesical BCG is best known as the most effective agent for the treatment of high-grade superficial bladder cancer [2-4]. In this context, BCG is used to reduce both the recurrence rate of bladder tumor and to diminish the risk of its progression [2,3]. As an adjunct to transurethral resection, BCG is the treatment of choice for urothelial carcinoma in-situ (CIS) and is commonly used for recurrent or multi-focal Ta and high grade T1 bladder lesions [5,6].

It is not clear how BCG alters the course of cystitis or cancer progression. Recently, however, the susceptibility to BCG was correlated with polymorphisms of the human NRAMP1 gene [7], providing interesting insights into the complexity of the genomics of BCG immunotherapy [8]. One theory is that intravesical BCG corrects an aberrant immune imbalance in the bladder, leading to long-term symptomatic improvement [1].

Here, we explore the possibility that BCG causes an extensive local inflammatory reaction in the bladder wall [9]. Of this, the massive appearance of cytokines in the urine of BCG-treated patients stands out [9]. Activated lymphocytes and macrophages are the most likely sources of these cytokines, but at present, other cellular sources such as urothelial cells cannot be ruled out [9]. BCG is internalized and processed by neutrophils [10], professional antigen-presenting cells, and urothelial tumor cells, resulting in altered gene expression and secretion of particular cytokines [9]. It was suggested that the effectiveness of BCG treatment is determined by two processes: an inflammatory one, followed by a delayed type of hypersensitivity response [11]. Others proposed three distinct phases in the immune response to BCG. In phase 1, BCG adheres to the urothelium via interaction between the bacterial antigen 85 complex and fibronectin [6,12] and urothelial cells. In addition to fibronectin, it has been suggested that toll-like receptors (TLRs) -2 and -4, present in immune cells, mediate BCG-induced immune responses [13-15]. Once internalized, BCG is processed both by professional antigen-presenting cells and urothelial cells, resulting in an altered gene expression [9]. This phase corresponds to the early release of so-called inflammatory cytokines (IL-1, IL-6, and IL-8 in humans) which may be responsible for certain adverse effects. Phase 2 consists of recognition of bacterial antigens by CD4 lymphocytes, which release mainly IL-2 and IFN-γ (TH_1 _response). This cell activation leads to phase 3, which consists of amplification of cytotoxic-populations: CD8 T cells, gamma-delta lymphocytes, macrophages, and natural killer (NK) cells. All these cells also release cytokines which then regulate BCG response [16].

More recently, studies have shown that mycobacterial DNA contains high amounts of CpG motifs. These CpG motifs induce tumor necrosis factor-related apoptosis-inducing ligand (TRAIL) expression [17] and increase serum levels of mouse keratinocyte-derived chemokine (KC), a functional homolog of human interleukin IL-8 [18]. Urinary TRAIL levels were initially undetectable in BCG therapy patients, but levels increased after subsequent treatments. More importantly, patients that responded to BCG therapy had significantly higher urine TRAIL levels, which killed bladder tumor cells *in vitro *versus non-responders. Given these data, it was proposed that TRAIL also plays a role in BCG-induced anti-tumor effects [17].

Nevertheless, BCG's mechanism of action remains poorly understood. Although systemic reactions have been reported, a likely scenario is that exposure to BCG results in a local immune response and massive inflammation [6], which makes it difficult to analyze the direct effects of BCG on the urinary bladder in BCG treated patients. Therefore, we propose to characterize in C57BL/6 mice, bladder responses to intravesical BCG therapy. C57BL/6 mice were chosen not because of the Th1 biased response of this strain [19], but because it will permit comparisons with other animal models of bladder inflammation already developed in C57BL/6 background [20,21].

In this first manuscript, we propose to determine whether the intact mouse urinary bladder takes up BCG, the time-dependent morphological alterations, and urinary cytokine release in response to intravesical BCG. These effects were compared to those elicited by other inflammatory stimuli (LPS and TNF-α) to differentiate BCG-specific effects from generalized inflammatory reactivity.

## Results

### BCG uptake by bladder urothelial cells

**Figure 1:**
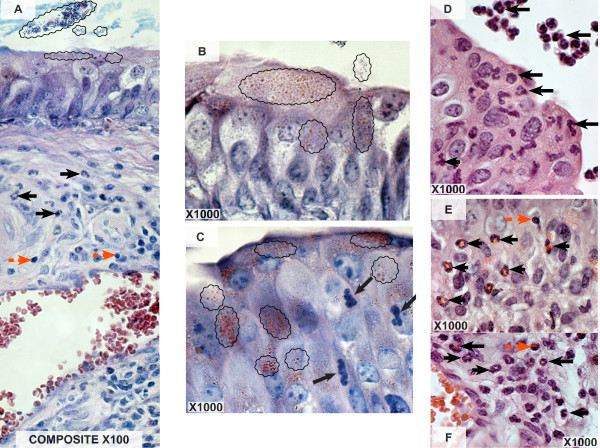
**BCG uptake by bladder urothelial cells. Representative photomicrographs indicating uptake of acid fast bacteria by the urothelial cells and infiltration of PMNs**. Twenty four hours after bladder instillation with BCG, bladders were removed and stained with fast acid (A-C) or H&E (D-F). Acid fast-positive bacteria were detected within umbrella cells and underlying urothelial cells, indicating that BCG is taken up by an apparent intact epithelium (Figure 1A-C). Black wavy lines indicate areas containing fast acid-positive bacteria; black arrows indicate PMNs, and red arrow indicates lymphocytes.

Twenty-four hours after instillation of BCG into the mouse bladder, fast acid-positive bacteria were detected within umbrella cells and underlying urothelial cells, indicating that BCG is taken up by an apparent intact epithelium (Figure 1A–C). The local inflammatory reaction in response to BCG was characterized by an initial increase in blood flow, enhanced vascular permeability characterized by edema, and an influx of different effector cells. At this time point, vasodilation was evident and inflammatory cells (PMNs and lymphocytes) populated the submucosal layer (Figure 1A). Interestingly, some intraepithelial PMNs were also observed nearby urothelial cells containing BCG (Figure 1C). PMNs invaded predominantly the sub-urothelial mucosal layer (Figure 1A and 1E), and some inflammatory cells were also observed outside the urothelium, in the bladder lumen (Figure 1D).

**Figure 2:**
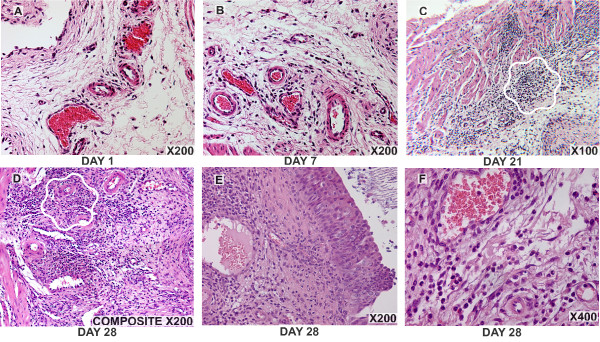
**Time-course of bladder morphological alterations in response to weekly instillations with BCG**. Representative photomicrographs of a time-dependent alteration of the mouse bladder secondary to intravesical instillation of BCG. Note, the intense edema observed days 1–7 after the first instillation with BCG (A and B) and the subsequent invasion of inflammatory cells into the submucosa at day 7 (B) and a time-dependent invasion of the detrusor muscle at days 21 (C) and 28 (D-F). Granuloma-like formation is indicated by wavy white lines.

**Figure 3:**
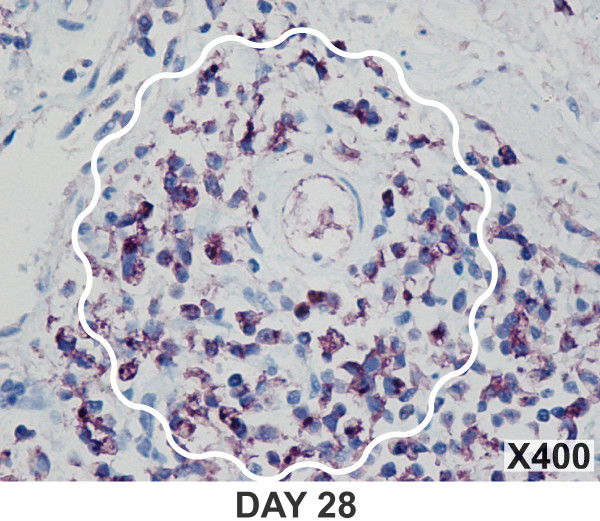
Granuloma-like formation is indicated by wavy white lines. The presence of monocyte/macrophages was determined by IHC immunohistochemistry with rat anti-mouse macrophage monocyte antibody and secondary antibody anti-mouse Fab-HRP (G).

In early time points, BCG induced an acute bladder inflammation characterized by a strong vascular component and edema. Characteristic photomicrographs of BCG-induced bladder inflammation exemplify the trend from an intense edema observed on days 1 and 7 (Figures 2A–B), and a gradual substitution of the edema by an influx of PMNs and other inflammatory cells (Figure 2C–F). Migrating inflammatory cells were initially observed in submucosa (Figure 2A–B) and gradually invaded the detrusor muscle (Figure 2C). From days 21 to 28, in addition to PMNs (Figure 2C–E), there was a substantial presence of monocyte-macrophages (Figure 3). During chronic inflammation, several spots presented granuloma-like formations [22] (Figure 2C and 3).

**Figure 4:**
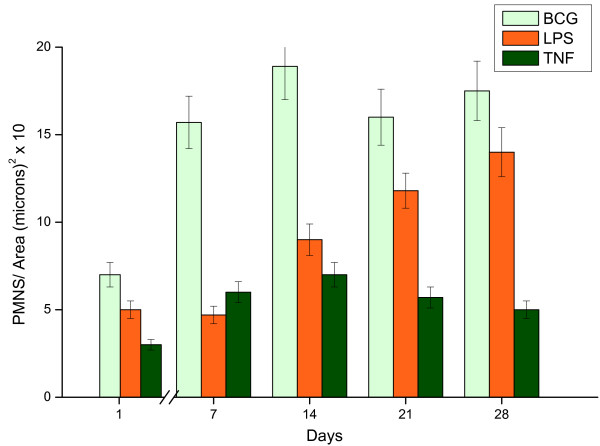
**PMNS. Mouse bladder morphological analysis in response to weekly instillations of BCG, LPS, and TNF-α**. The time-course of inflammation was characterized by the number of polymorphonuclear leukocytes [PMNs] infiltrating the urinary bladder.

**Figure 5:**
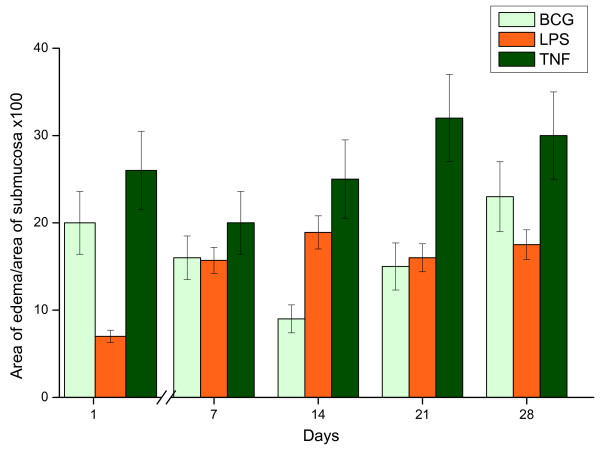
**Edema. Mouse bladder morphological analysis in response to weekly instillations of BCG, LPS, and TNF-α**. The time-course of inflammation was characterized by the edema in the urinary bladder.

All three stimuli induced bladder inflammation. BCG was the strongest stimulus for PMN migration (Figure 4), whereas edema appeared as an acute response to all stimuli (Figure 5). Interestingly, the area of edema formation in response to BCG was biphasic, appearing as early as twenty four hours after BCG instillation and declining at fourteen days despite weekly instillations with BCG (Figure 5).

Next, we determined how long the inflammatory response to BCG was maintained. For this purpose, mice that received weekly instillations of BCG for 4 weeks had the treatment discontinued (day zero), and were examined weekly up to 5 weeks for the presence of bladder inflammation. The degree of inflammation observed at the end of 4 weekly BCG instillations (day zero) was maintained up to three weeks following discontinuation of the therapy. Both the edema and PMNs were reduced at 4 weeks whereas the vasodilation was maintained for the whole 5 weeks of observation (data not shown).

Together, these results indicate that bladder instillation with BCG or TNF-α induce a classical inflammatory response, as previously described for LPS [23,24]. However, relative to LPS and TNF-α, bladder responses to BCG produce a higher PMN influx, with cells remaining well after discontinuation of the treatment. Additionally, BCG was the only agent capable of inducing formation of granuloma-like structures.

### Urinary cytokine levels

**Figure 6:**
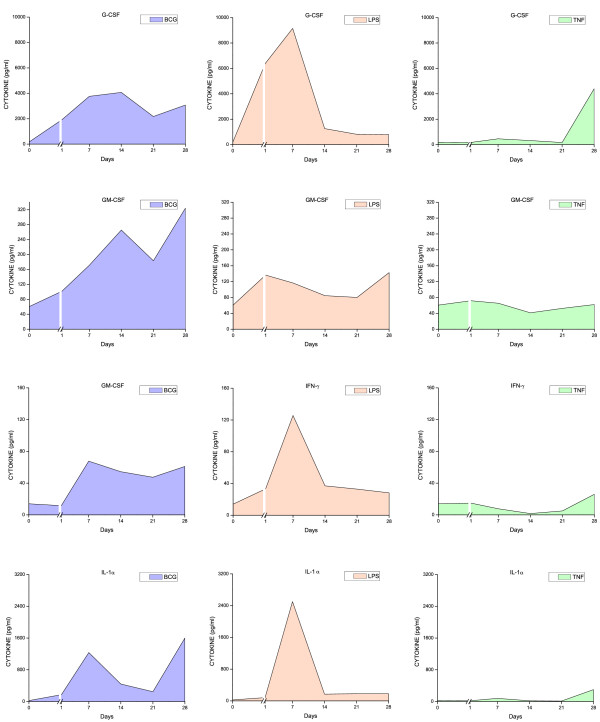
**Urinary cytokine levels (pg/ml) in response to weekly instillations of BCG, TNF-α, and LPS into the bladder of female mice**. Cytokine measurements were performed using fifty microliters of each sample and done in duplicate on the Luminex 100 (Bio-Rad Laboratories, Hercules, CA) using a Bio-Plex Mouse Cytokine 18-Plex (catalog #171-F11181, BioRad). The concentrations of analytes in these assays were quantitated using a standard curve. A regression analysis was performed to derive an equation that was then used to predict the concentration of the unknown samples. Time zero indicates basal levels of cytokines before instillation of any agent. A break at day 1 was added to each graph to contrast the altered cytokine levels when compared to basal levels.

**Figure 7:**
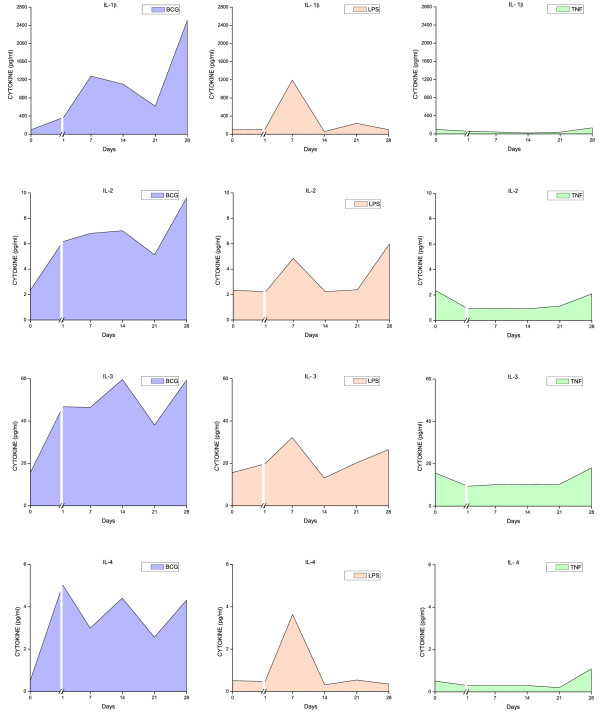
**Urinary cytokine levels (pg/ml) in response to weekly instillations of BCG, TNF-α, and LPS into the bladder of female mice**. Cytokine measurements were performed using fifty microliters of each sample and done in duplicate on the Luminex 100 (Bio-Rad Laboratories, Hercules, CA) using a Bio-Plex Mouse Cytokine 18-Plex (catalog #171-F11181, BioRad). The concentrations of analytes in these assays were quantitated using a standard curve. A regression analysis was performed to derive an equation that was then used to predict the concentration of the unknown samples. Time zero indicates basal levels of cytokines before instillation of any agent. A break at day 1 was added to each graph to contrast the altered cytokine levels when compared to basal levels.

**Figure 8:**
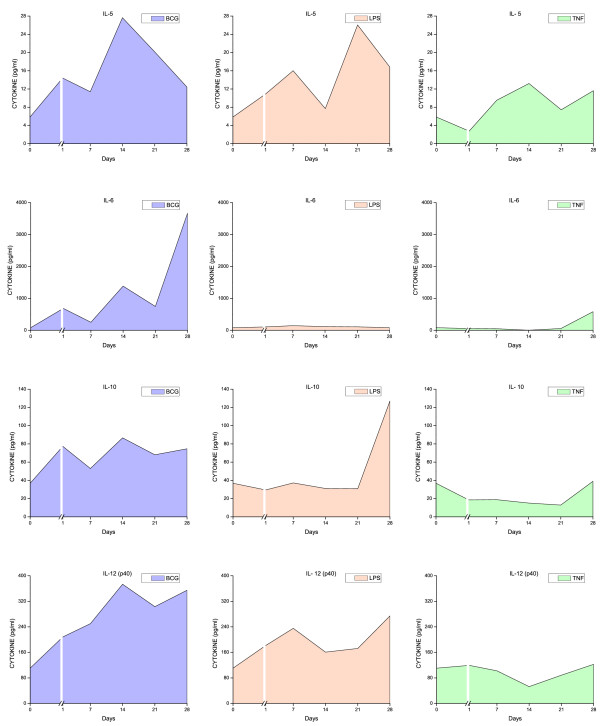
**Urinary cytokine levels (pg/ml) in response to weekly instillations of BCG, TNF-α, and LPS into the bladder of female mice**. Cytokine measurements were performed using fifty microliters of each sample and done in duplicate on the Luminex 100 (Bio-Rad Laboratories, Hercules, CA) using a Bio-Plex Mouse Cytokine 18-Plex (catalog #171-F11181, BioRad). The concentrations of analytes in these assays were quantitated using a standard curve. A regression analysis was performed to derive an equation that was then used to predict the concentration of the unknown samples. Time zero indicates basal levels of cytokines before instillation of any agent. A break at day 1 was added to each graph to contrast the altered cytokine levels when compared to basal levels.

**Figure 9:**
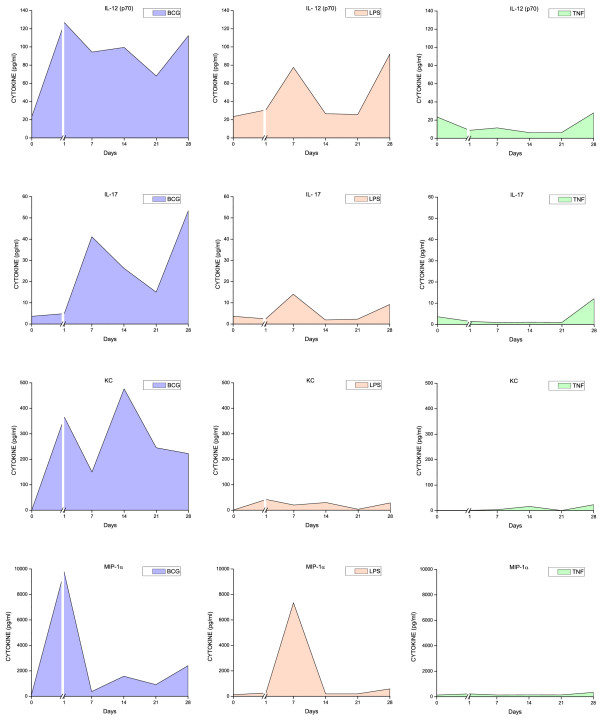
**Urinary cytokine levels (pg/ml) in response to weekly instillations of BCG, TNF-α, and LPS into the bladder of female mice**. Cytokine measurements were performed using fifty microliters of each sample and done in duplicate on the Luminex 100 (Bio-Rad Laboratories, Hercules, CA) using a Bio-Plex Mouse Cytokine 18-Plex (catalog #171-F11181, BioRad). The concentrations of analytes in these assays were quantitated using a standard curve. A regression analysis was performed to derive an equation that was then used to predict the concentration of the unknown samples. Time zero indicates basal levels of cytokines before instillation of any agent. A break at day 1 was added to each graph to contrast the altered cytokine levels when compared to basal levels.

**Figure 10:**
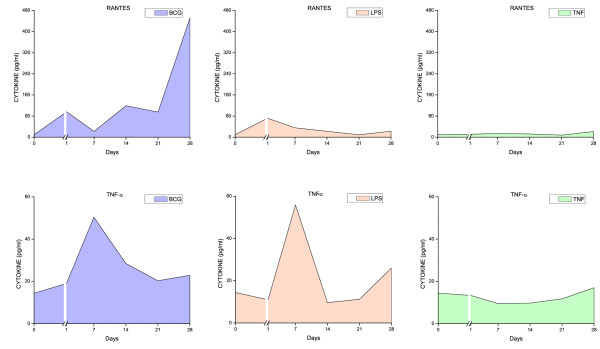
**Urinary cytokine levels (pg/ml) in response to weekly instillations of BCG, TNF-α, and LPS into the bladder of female mice**. Cytokine measurements were performed using fifty microliters of each sample and done in duplicate on the Luminex 100 (Bio-Rad Laboratories, Hercules, CA) using a Bio-Plex Mouse Cytokine 18-Plex (catalog #171-F11181, BioRad). The concentrations of analytes in these assays were quantitated using a standard curve. A regression analysis was performed to derive an equation that was then used to predict the concentration of the unknown samples. Time zero indicates basal levels of cytokines before instillation of any agent. A break at day 1 was added to each graph to contrast the altered cytokine levels when compared to basal levels.

All three stimuli induced a time-dependent increase in urinary cytokine levels (Figures 6, 7, 8, 9, and 10). Overall, BCG induced the most comprehensive cytokine responses, followed by LPS and TNF-α. Additionally, some cytokine levels were altered acutely, whereas others were elevated only after chronic stimulation (detailed below). Cytokine induction relative to baseline levels was calculated and ranked for each inflammatory agent (Table 2). Consistent with the rapid and massive neutrophil infiltrates observed histologically, relative induction of KC was highest in response to BCG, reaching 47,000 times the control level. In addition to KC, BCG induced the release of: MIP-1α, IL-1α, RANTES, IL-6, G-CSF, IL-1β, IL-17, IL-4, IL-12(p70), and GM-CSF. In contrast to BCG, the unique cytokine induced by LPS was IL-1β, and MIP-1α reached the highest peak in response to acute LPS (Table 2). In addition to MIP-1α, LPS induced the release of the following cytokines (listed in order of induction levels): IL-1α, G-CSF, KC, IL-1β, IFNγ, RANTES, IL-4, IL-5, IL-12 (p70), TNFα, IL-17, and IL-10. Finally, G-CSF was found to be the highest cytokine in response to TNF-α. In addition to G-CSF, TNF-α induced the following cytokines (listed in order of induction levels): KC, IL-1α, IL-6, IL-2, IL-17, MIP-1α, IL-5, RANTES, IL-4, IFN-γ, and IL-1β (Table 2).

**Table 2:** Peak urinary cytokine levels in response to BCG, TNF, and LPS calculated as percent of baseline

**BCG**		**TNF**		**LPS**	
KC	47474	G-CSF	2424	MIP-1a	732446
MIP-1a	7314	KC	2305	IL-1a	10048
IL-1a	6422	IL-1a	1210	G-CSF	5045
RANTES	4732	IL-6	729	KC	4186
IL-6	4591	IL-2	410	Il-1b	1064
G-CSF	2244	IL-17	334	IFN-g	902
Il-1b	2236	MIP-1a	251	RANTES	751
IL-17	1469	Il-5	226	IL-4	716
IL-4	991	RANTES	226	Il-5	446
IL-12(p70)	540	IL-4	214	IL-12(p70)	393
GM-CSF	533	IFN-g	186	TNF-a	387
IFN-g	485	Il-1b	120	IL-17	387
Il-5	473	IL-12(p70)	119	IL-10	345
IL-2	410	GM-CSF	118	IL-2	255
IL-3	382	TNF-a	118	IL-12(p40)	248
TNF-a	349	IL-3	115	GM-CSF	235
IL-12(p40)	338	IL-12(p40)	111	IL-3	206
IL-10	235	IL-10	106	IL-6	173

Both pro-inflammatory and anti-inflammatory cytokines were detected in the mouse urine following inflammation induced by BCG, TNF-α, and LPS. However, IL-17 was induced primarily in response to BCG (Figure 9). In addition, we confirmed previous reports indicating that certain cytokines, such as IL-12 represented the early responses of the urinary bladder to BCG [25]. In the present study, this cytokine was the first to be released and may reflect a direct stimulation of bladder resident cells, whereas cytokines induced subsequent to this acute-response phase may be the result of secondary mediator synthesis by migrating inflammatory cells. In addition to IL-12, other cytokines, like GM-CSF and IL-2, represent an early response to all three agents.

To better characterize BCG-specific responses, the time-courses of cytokine levels were mathematically modeled, and time-dependent inductions of the 3 agents compared. The time-course of IL-1β, IL-2, IL-3, IL-4, IL-6, IL-10, IL-12p70, IL-17, GM-CSF, KC, and RANTES induction discriminate between the effects of BCG relative to the inflammatory responses obtained with LPS and TNF-α (Table 3).

**Table 3:** Discriminators of BCG effects versus TNF and LPS over time.

**Analyte**	**p value**
GM-CSF	0.001
IL-1 beta	0.007
IL-2	0.006
IL-3	0.032
IL-4	0.016
IL-6	0.000
IL-10	0.001
IL-12p70	0.012
IL-17	0.002
KC	0.018
RANTES*	0.001

Urinary cytokines response over time was determined using a mixed modeling approach, according to the following equation: Cytokine Level = (a) * Time + (b) Trx * Time, where Trx is BCG vs LPS & TNF-Alpha. A variance component covariance matrix was used to account for the correlation structure inherent in longitudinal measurements of cytokines.*Time squared interaction.

**Figure 11:**
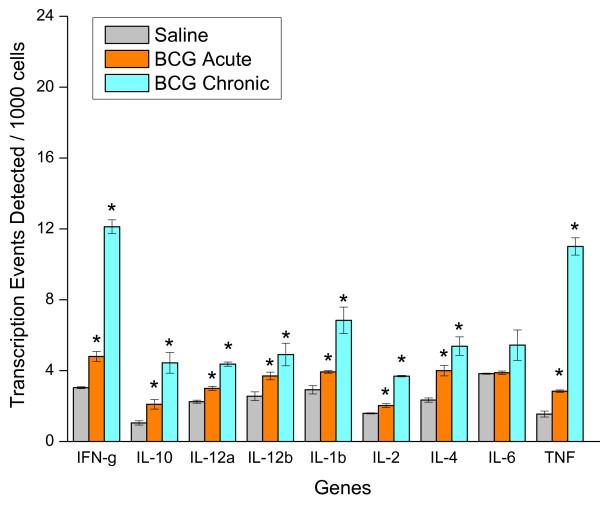
**Chromatin immunoprecipitation (CHIP)/quantitative real-time polymerase chain reaction (Q-PCR)-Based Assays**. Female C57BL/6J mice were anesthetized and instilled with 200 μl of one of the following substances: BCG (TheraCys^®^-Aventis-Pasteur; total dose of 1.35 mg) or pyrogen-free saline on days 1, 7, 14, and 21, as described in methods. Mice were euthanized 24 hours after a single instillation (**BCG Acute**) or 7 days after 4 weekly instillations (**BCG Chronic**). A total of 60 mice were used (20 mice per group). Bladders were removed rapidly, frozen, and shipped to Genpathway  for querying the chromatin for transcription of listed genes (Genpathway's TranscriptionPath Query assay). After isolation, the chromatin was incubated with an antibody against RNA polymerase II (Abcam) to precipitate the DNA transcriptome. The final CHIP DNAs were then used as templates in Q-PCR reactions using specific primer pairs (Table Table 1). Results are presented as average and standard error of Transcription Events Detected Per 1000 Cells. Results were grouped to facilitate their visualization. Cytokines. Graphics illustrated in figures 11, 12, and 13 present the same X-axis to permit comparisons between gene families.

**Figure 12:**
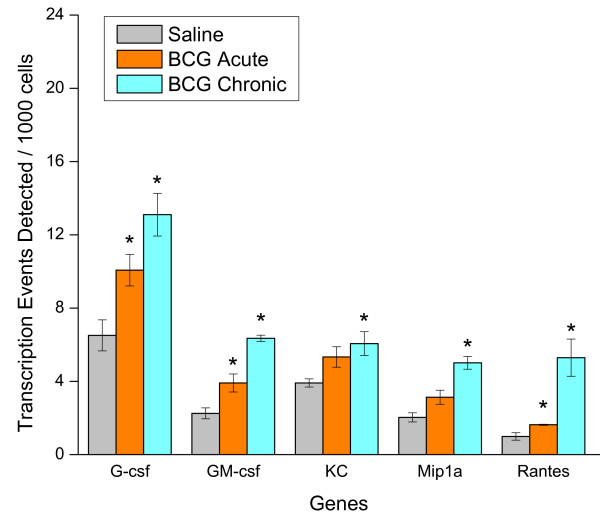
**Chromatin immunoprecipitation (CHIP)/quantitative real-time polymerase chain reaction (Q-PCR)-Based Assays**. Female C57BL/6J mice were anesthetized and instilled with 200 μl of one of the following substances: BCG (TheraCys^®^-Aventis-Pasteur; total dose of 1.35 mg) or pyrogen-free saline on days 1, 7, 14, and 21, as described in methods. Mice were euthanized 24 hours after a single instillation (**BCG Acute**) or 7 days after 4 weekly instillations (**BCG Chronic**). A total of 60 mice were used (20 mice per group). Bladders were removed rapidly, frozen, and shipped to Genpathway  for querying the chromatin for transcription of listed genes (Genpathway's TranscriptionPath Query assay). After isolation, the chromatin was incubated with an antibody against RNA polymerase II (Abcam) to precipitate the DNA transcriptome. The final CHIP DNAs were then used as templates in Q-PCR reactions using specific primer pairs (Table 1). Results are presented as average and standard error of Transcription Events Detected Per 1000 Cells. Results were grouped to facilitate their visualization. Chemokines. Graphics illustrated in figures 11, 12, and 13 present the same X-axis to permit comparisons between gene families.

**Figure 13:**
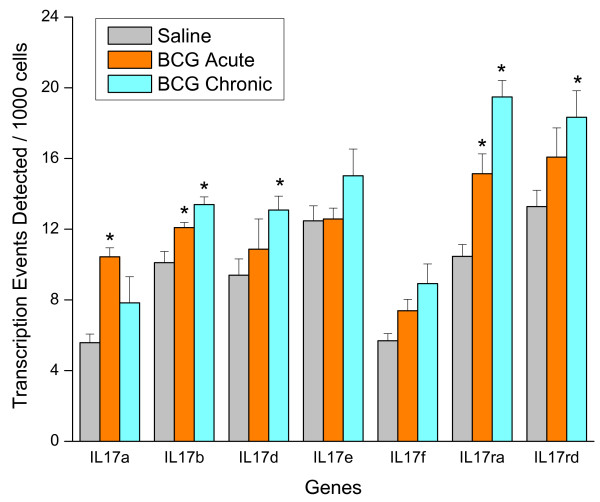
**Chromatin immunoprecipitation (CHIP)/quantitative real-time polymerase chain reaction (Q-PCR)-Based Assays**. Female C57BL/6J mice were anesthetized and instilled with 200 μl of one of the following substances: BCG (TheraCys^®^-Aventis-Pasteur; total dose of 1.35 mg) or pyrogen-free saline on days 1, 7, 14, and 21, as described in methods. Mice were euthanized 24 hours after a single instillation (**BCG Acute**) or 7 days after 4 weekly instillations (**BCG Chronic**). A total of 60 mice were used (20 mice per group). Bladders were removed rapidly, frozen, and shipped to Genpathway  for querying the chromatin for transcription of listed genes (Genpathway's TranscriptionPath Query assay). After isolation, the chromatin was incubated with an antibody against RNA polymerase II (Abcam) to precipitate the DNA transcriptome. The final CHIP DNAs were then used as templates in Q-PCR reactions using specific primer pairs (Table 1). Results are presented as average and standard error of Transcription Events Detected Per 1000 Cells. Results were grouped to facilitate their visualization. IL-17 family. Graphics illustrated in figures 11, 12, and 13 present the same X-axis to permit comparisons between gene families.

Next, we used CHIP/Q-PCR to investigate whether acute BCG (24 hours after a single instillation) and chronic BCG (7 days after four weekly instillations) would also alter cytokine gene expression in the whole mouse bladder. With the exception of IL-6, KC, and MIP1α, acute BCG instillation induced an increased expression of all other cytokine and chemokine genes tested (Figures 11 and 12). Chronic BCG instillation induced increased expression of all cytokine genes measured with the exception of IL-6 (Figures 11 and 12). In addition, particular attention was paid to the IL-17 gene family since IL-17A protein was one of the cytokines that discriminated between BCG and the other pro-inflammatory stimuli (Table 3). CHIP/Q-PCR results indicate a differential up regulation of this family between acute and chronic BCG (Figure 13).

## Discussion

### BCG uptake by bladder urothelial cells

Our results indicate that BCG is taken up by an apparent intact urothelium and accumulates within urothelial cells. Others have shown that *E. coli *is present in pod-like bulges on the urothelial bladder surface, which represents a reservoir for bacteria and explains the persistence of bladder infections [26]. It remains to be determined whether this mechanism applies to BCG. Nevertheless, the intact mouse urothelium is highly BCG responsive and, as such, is a useful model to study mechanisms and receptors that modulate the BCG uptake by the urinary bladder. In this context, previous results from our laboratory indicate that LPS-fluorescein is taken up by the intact mouse bladder urothelium, provokes bladder inflammation, and it is systemically distributed [23]. Together, our findings indicate that although the urothelium present a tightly controlled permeability, certain bacteria and toxins find their way to penetrate this layer and to cause inflammation.

### Morphological alterations – PMNs infiltration

Our results indicate that bladder inflammation is part of the response to intravesical BCG therapy (Figures 1, 2, 3). This inflammation is characterized by a rapid onset of edema and a graded and massive migration of inflammatory cells (lymphocytes, PMNs and monocytes/macrophages) initially into the lamina propria, which spreads to detrusor muscle as well (Figure 1). In humans, intravesical therapy induces a well-described T-lymphocyte predominant inflammatory infiltrate in the bladder wall [27,28], and it is proposed that helper and cytotoxic T cells and, most probably, natural killer cells are necessary for anti-tumor effects and the Th-1 biased immune response that underlines BCG therapy [29]. Although BCG-treated mouse bladder also exhibited lymphocytic infiltrates during the acute response and macrophages during the late phase responses (Figure 3), PMNs were overwhelmingly present, suggesting the initial response is dominated by these cells. Indeed, our histological results (Figure 1) and the consequent morphological analysis (Figure 4) suggest that PMNs are of prime importance in the model under investigation. This cell-type is a bona fide inflammatory cell, and it accounts for more than 90% of the urinary cellular infiltrate early after BCG-mediated immune responses. Indeed, Siracusano et al confirmed that in humans, following intravesical BCG prophylaxis, a large number of PMNs transmigrated through the urothelium and are found isolated or adherent to detached urothelial cells in the urine [30]. In addition, flow cytometry analysis of fresh urine from patients receiving intravesical BCG revealed TRAIL-expressing neutrophils [17]. Others have shown in mice that PMNs migrate into the bladder after intravesical BCG instillation, and that depletion of PMNs from tumor-bearing mice completely abrogated antitumor efficacy of BCG [31]. In vivo, depletion of PMNs from BCG-treated mice significantly impaired CD4(+) T-cell trafficking to the bladder [31]. Furthermore, PMNs isolated from the urine of BCG-treated patients were a major source of IL-8, growth-related oncogene-alpha, MIP-1α, and inflammatory cytokine migration inhibitory factor [31]. Therefore, our results agree with other findings indicating that PMNs direct the migration of effector cells to the bladder and are indispensable for effective tumor immunotherapy [17,30,31].

Subsequent to the initial response, macrophages localized in close proximity to neutrophils in a granuloma-like structure that occurred in the mouse bladder over time (Figure 3). The granuloma is a hallmark of Mycobacterium-induced pathology [22,32,33]. BCG-induced granuloma seems to depend on TNF-α release [34,35]. Indeed, transgenic mice expressing high serum levels of sTNFR1, capable of neutralizing all circulating TNF, failed to develop differentiated granulomas and bactericidal mechanisms, and succumbed to BCG infection [36]. However, in our studies, chronic bladder instillation with TNF-α alone was not enough to induce granuloma formation. The latter could be due to a reduced uptake of TNF-α by the urinary bladder, or that other factors are necessary for the development of granuloma. Indeed, recent results indicate that granuloma formation depends primarily on the presence of transmembrane TNF-α [35].

Although systemic reactions (evolution of cellular immune response or systemic production of cytokines and oxygen free radicals) have been reported in response to bladder instillation with BCG, a likely scenario is that exposure to BCG results in a massive local inflammatory response [37]. Correspondingly, in the present mouse model, BCG induces a stronger PMN infiltration than TNF-α and LPS. Additionally, granuloma formation was only observed in response to chronic BCG instillation.

### LPS and TNF as pro-inflammatory stimuli

The rationale for the use of LPS was that the bladder inflammatory responses to this bacterial toxin have been well characterized in our laboratory. We have presented evidence that LPS induces a time-dependent cystitis [24] concomitantly with the release of cytokines [23]. In contrast, although there is strong circumstantial evidence indicating that TNF-α plays a fundamental role in bladder inflammation [21], to our knowledge, the present study is the first to use intravesical TNF-α as a model for cystitis. TNF-α, at the concentration used in this study, was the weakest inducer of cytokine release (Figures 6, 7, 8, 9, 10). Although the concentration of TNF-α employed in this study was at least 100-fold higher than the one used to provoke neutrophil migration in the mouse skin [38], it is possible that much higher concentrations of TNF-α would lead to levels of urinary cytokines comparable to LPS. In the context of TNF-α, it seems that the regimen applied in this study, allowing at least 7 days between bladder instillation and urine collection, decreased the possible bias of TNF-α instillation on its urinary levels.

### BCG-induced cytokine release

Keratinocyte-derived chemokine (KC; CXCl1; or Gro-α) levels were the most altered by BCG (Table 2). As the mouse does not produce IL-8, measurement of mouse KC, a functional homolog of human IL-8 [18], could give us some information regarding a role of this pro-inflammatory cytokine in mediating BCG responses. KC is a potent neutrophil chemoattractant that can be secreted by neutrophils, epithelial cells, endothelial cells, smooth muscle cells, fibroblasts, macrophages, platelets, and lymphocytes [39]. The main stimuli for KC production are IL-1, TNF-α, bacterial products, radical oxygen species, and LPS, as well as the T cell products, such as IL-4 and IFN-γ [39].

IL-17 was significantly increased by BCG and only slightly altered by long term treatment with LPS or TNF-α. IL-17 belongs to a family of pro-inflammatory cytokines [40,41]. IL-17 is a CD4 T cell-derived pro-inflammatory and pro-angiogenic cytokine that further promotes IL-6 production and VEGF-mediated angiogenesis [42]. IL-17 is also involved in Fas ligand-induced inflammation [43]. Among the members of this family, IL-17A is the most studied, and it is secreted by activated T cells and acts on stromal cells to produce pro-inflammatory cytokines, such as GM-CSF, G-CSF, CXCL1, and CXCL8 (IL-8) [44]. IL-17 is a strong activator of neutrophil migration and infiltration, although perhaps not directly [44,45]. To our knowledge, this is the first report of an increase in urinary IL-17 release and gene expression in response to intravesical BCG.

These findings suggest that the therapeutic effects of BCG are due in part to the rapid accumulation of antigen presenting, and activated immune cells that are responsible for the production of a multiphasic immune response as demonstrated by the presence of TH1 (IFN-γ, IL-1, IL-12, and TNF-α), TH2 (IL-4, IL-5, IL-6, and IL-10), and TH17 (IL-17 and IL-6) cytokines.

### Time-course of cytokine release

Because of the innumerous side-effects of BCG therapy, other authors have determined the effect of different preparations [46] and regimens of intravesical BCG instillations in mice on the dynamics of Th1/Th2 cytokines [19,47]. It was suggested that the first BCG instillation is fundamental for the overall response [19]. In the present study, cytokine levels in the urine were elevated as early as 1 day after BCG instillation. The earliest cytokines induced by BCG included: IL-12 (p70 and p40), IL-2, IL-3, IL-4, MIP-1α, IL-10, and KC (Figures 6, 7, 8, 9, 10). Those cytokines were released when inflammatory cell infiltrates were minimal (Figure 2A–B), and may reflect a direct effect of BCG on resident cells, such as urothelial cells and fibroblasts. Indeed, BCG stimulates IL-12 release by human urothelial cells in culture concomitantly with up regulation of toll-like receptors -2 and -4 [15]. Of interest, O'Donnel and collaborators have shown that IL-12 expression preceded the other cytokines [25]. In the present work, IFN-γ, a major Th1 cytokine, appeared in the urine late in response to BCG (Figures 6, 7, 8, 9, 10). In humans, IFN-γ was barely detectable after the first two BCG instillations, but gradually increased from the third instillation onwards [48]. The peak weekly cytokine response per patient usually occurred between the fourth and sixth treatment for IFN-γ [49]. Others have found IFN-γ to be a late cytokine in response to BCG, and its induction requires involvement of various endogenously produced Th1 and Th2 cytokines.

Among the cytokines released by BCG, attention has been paid to IL-6 because its urinary levels are correlated with symptom scores in cystitis [50,51] and are reduced by BCG therapy in bladder cancer [52]. However, our CHIP/Q-PCR results did not demonstrate a significant increase in IL-6 gene following acute and chronic BCG.

Regarding the anti-inflammatory cytokines, we found that BCG also induces the release of IL-10 as early as 1 day after the first instillation. Others have found that BCG also releases IL-10 from the mouse bladder [37]. IL-10 is an important down-regulator of delayed-type hypersensitivity (DTH). In addition, in mouse studies, the absence of IL-10 abrogated either by antibody inhibition or the use of genetically modified, IL-10 deficient mice, resulted in enhanced DTH responses [37].

Together, our results demonstrate a tractable approach for assessing the complex cytokine networks induced by BCG during both acute and chronic stages. This approach also provided the means to make relatively rigorous comparisons of the BCG response to other pro-inflammatory stimuli. Our analysis revealed that IL-1β, IL-2, IL-3, IL-4, IL-6, IL-10,IL-17, GM-CSF, KC, and Rantes were the best discriminators of the BCG response relative to TNF-α and LPS. Although the levels of IL-2 and IL-4 at a given time point were relatively low, our mathematical model takes into consideration the cytokines over time rather than their individual values. Using this approach, both IL-2 and IL-4 were included as discriminators for the BCG responses because of their significant *p *values (Table 3).

We went further to determine whether acute and chronic BCG treatment could alter bladder gene expression. For this purpose, we examined 2 groups of mice that received BCG intravesicaly, as described in methods. The acute group was examined 24 hours after a single instillation and a chronic group was examined 7 days after 4 weekly instillations. We used a combination of CHIP assay and Q-PCR which has the advantage to use a quantitative method to assess genes of interest in the bladder transcriptome. This assay takes into consideration genes that are actively transcribed in contrast to cDNA array technologies that queries RNA accumulation. The results obtained in the acute group (24 hours following a single BCG instillation) indicate that with the exception of IL-6, KC, and MIP1α, there was a significant increase in all other RNA encoding cytokines and chemokines. In contrast, the RNA accumulation in consequence to chronic BCG instillation had a strong correlation with the levels of cytokines and chemokines and the increased urinary levels of these proteins. The disadvantage of the CHIP/Q-PCR method is the amount of chromatin necessary for CHIP, which limits this analysis to the whole bladder and not the specific layers. Our CHIP/Q-PCR results have to be taken in the context that migrating inflammatory cells, in addition to resident cells, contributed to the measured values. Future studies using individual bladder layers such as the urothelium, submucosa, and detrusor muscle should provide further detail on the source of urinary cytokines.

## Conclusion

BCG induces a unique type of persisting bladder inflammation different from TNF-α, LPS, and, most likely other classical pro-inflammatory stimuli. Among the different cytokines, this study highlighted the importance of IL-1β, IL-2, IL-3, IL-4, IL-6, IL-10, IL-17, GM-CSF, KC, and Rantes as discriminators between overall inflammation and bladder responses to BCG instillation. To the best of our knowledge, the present work is the first to report that BCG induces an increase in the IL-17 family genes.

## Materials and methods

### Animals

Ten to twelve week-old C57BL/6 female mice (The Jackson Laboratory; Bar Harbor, ME) were anesthetized, transurethrally catheterized as previously described [21,24,53,54], and instilled with 200 μl of one of the following substances: BCG (TheraCys^®^-Aventis-Pasteur; total dose of 1.35 mg [19]), *E. coli *LPS (strain 055:B5; 100 μg/ml [23,24,55]), TNFα (1 μg/ml), or pyrogen-free saline on days 1, 7, 14, and 21.

The urine was collected for cytokine analysis at time points indicated below (n = 6 mice per group), and the urinary bladder was removed for morphometric analysis (n = 8). All animal experimentation described here was performed in conformity with the "Guiding Principles for Research Involving Animals and Human Beings" (OUHSC Animal Care & Use Committee protocols #05-088 and 05-081).

### Histological Analyses

Bladders were fixed in 4% buffered formaldehyde, embedded in paraffin, cut serially into four 5-μm sections (8 μm apart), and subsequently stained with hematoxylin and eosin (H&E) and Ziehl-Neelsen (acid fast) [36]. Macrophages were visualized using immunohistochemistry with rat anti-mouse macrophage monocyte antibody (MCA519G, Serotec LTD, Oxford, UK) and secondary antibody anti-mouse Fab-HRP, as previously described [24]. Bladder stained sections were visualized under microscope (Eclipse E600, Nikon, Lewisville, TX). All tissues were photographed at room temperature by a digital camera (DXM1200; Nikon). Exposure times were held constant when acquiring images from different groups.

### Morphometric analysis of inflammation

Images were analyzed with Image-Pro Analyzer^® ^(Media Cybernetics Inc.; Silver Spring, MD). The number of polymorphonuclear [PMNs] leukocytes was the most reproducible sign of acute bladder inflammation and, therefore, it was used for quantification. PMNs were counted in a blinded fashion in 5 random fields per slide in two non-consecutive sections per urinary bladder at 200× magnification. The number of PMNS was normalized per cross-sectional area (μm^2^). In addition, the area of edema was determined from the same cross sections and normalized per cross-sectional area of the mucosa/submucosa.

### Urine collection

Urine for cytokine analysis was collected during early morning hours at the following time points: time 0 (previous to first instillation to determine basal release), one and seven days after a single bladder instillation, and seven days after each subsequent intravesical instillation performed at days 7, 14, and 21. A sample of urine from each mouse was transferred to a test tube and stabilized by the addition of an equal volume of a 10-fold concentrated buffer containing 2 M Tris-HCl (pH 7.6), 5% BSA, 0.1% sodium azide, and the following protease inhibitors: aprotinin, pepstatin, leupeptin at 0.01 mg/ml, and AEBSF, 4-(2-aminoethyl) benzenesulfonyl fluoride at 0.1 mg/ml, as described by O'Donnell et al [25]. Samples were then stored at -70C for batch cytokine analysis.

### Urinary Cytokines

Buffered urine samples were thawed and fifty microliters of each sample were analyzed in duplicate on the Luminex 100 (Bio-Rad Laboratories, Hercules, CA) using a Bio-Plex Mouse Cytokine 18-Plex (catalog #171-F11181, BioRad). The Bio-Plex suspension array system, which incorporates a novel technology using color-coded microspheres, permits the simultaneous detection of 18 cytokines and chemokines in a single well of a 96-well microplate. Cytokines measured included interleukin-1β (IL-1β), IL-1α, IL-2, IL-3, IL-4, IL-5, IL-6, IL-10, IL-12 (p40), IL-12 (p70), IL-17, granulocyte colony factor (G-CSF), granulocyte macrophage colony stimulating factor (GM-CSF), interferon-γ (IFN-γ), tumor necrosis factor-α(TNF-α), keratinocyte-derived chemokine (KC), CCL3 [macrophage inflammatory protein-1α (MIP-1α)], and CCL5 [regulated upon activation, normal T cell expressed and secreted (RANTES)]. A broad sensitivity range of standards (Biosource International, Camarillo, CA) ranging from 1.95 to 32000 pg/ml were used to help enable the quantitation of a dynamic wide range of cytokine concentrations, and to provide the greatest sensitivity. The concentrations of analytes in these assays were quantitated using a standard curve. A 4 parameter logistic regression was performed to derive an equation that was then used to predict the concentration of the unknown samples.

### Chromatin immunoprecipitation (CHIP) quantitative real-time polymerase chain reaction (Q-PCR)-Based Assays

To determine whether intravesical BCG treatment would alter bladder cytokine gene expression, we used chromatin immunoprecipitation combined with Q-PCR. For this purpose, female C57BL/6J mice were anesthetized and instilled with 200 μl of one of the following substances: BCG (TheraCys^®^-Aventis-Pasteur; total dose of 1.35 mg) or pyrogen-free saline on days 1, 7, 14, and 21, as described above. Mice were euthanized with pentobarbital (200 mg/kg, i.p.) 24 hours after a single instillation (**BCG acute**) or 7 days after 4 weekly instillations (**BCG chronic**). A total of 60 mice were used (20 mice per group). Bladders were removed rapidly, frozen, and were shipped to Genpathway [56] for querying the chromatin for gene transcription (Genpathway's TranscriptionPath Query assay) [57].

The urinary bladders were exposed briefly to formaldehyde for cross-linking of the proteins and DNA together, followed by sonication to fragment the DNA into pieces of approximately 300–500 base pairs. An antibody against RNA polymerase II (Abcam) was then used to precipitate the DNA transcriptome. The Ab-protein-DNA complexes were purified using beads coupled to protein A. The DNA was isolated from the complexes using a combination of heat to reverse cross-linking, RNase and proteases, and then purified using phenol extraction and EtOH precipitation. The final CHIP DNAs were then used as templates in quantitative PCR reactions using primer pairs specific for each gene of interest. Quantitative PCR was carried out using Taq polymerase (iQ SYBR Green Supermix, Bio-Rad). Primer pairs were designed using Primer 3 [58]. Details of the primer sequences are given in Table 1. The designed primers shared 100% homology with the target sequence but no significant homology with other sequences.

**Table 1:** Q-PCR PRIMERS

*ACCESSION NUMBER*	*SYMBOL*	*FORWARD PRIMER*	*REVERSE PRIMER*
NM_009971	G-csf	tgtcctcacaggaacgaagtc	cttcctcctgcctcctctg
NM_009969	GM-csf	gccacagttggaaggcagta	aaatataatggtccctatcagtagaaa
NM_008337	IFNg	agacttctgggcgtcctacc	tgaccaagcctttcataagatg
NM_010548	IL-10	gctgagactttcgctcctctc	agctccaaggcacctgttc
NM_008351	IL-12a	attgccacggtttcctctc	cctccttccctcctctgttc
NM_008352	IL-12b	tcaccttctctgggcatttc	gctattgggcatgcagtgag
NM_008361	IL-1b	ttcccaatccctcaacagtc	atgttctggagcaggcagtg
NM_008366	IL-2	acaatgtgggtgggtcactg	tgtcaagatagccaggaagacac
NM_021283	IL-4	ggctttcctctttcccactc	agccgccatgagagctaag
NM_031168	IL-6	gctgggattcttcaccactg	tgacttgtcctgagacctgatg
NM_008176	KC (Cxcl1)	tcgtctttcatattgtatggtcaac	cgagacgagaccaggagaaac
NM_011337	Mip1a (Ccl3)	ctccctcccagttggtcac	ttaaagggcatatttattacttctctg
NM_013653	Rantes (Ccl5)	cctattatgagccggcagag	gggtgccgtagatcattctg
NM_013693	TNFa	tcaggttgcctctgtctcag	gctctgtgaggaaggctgtg
NM_010552	IL17a	tcatttcctcctggcttttg	tgagcttcccagatcacaga
NM_019508	IL17b	ctttccccactctcccagac	caaccaacccaacgccttac
NM_145837	IL17d	ggccaaaacacacacaggta	ccttggtcagaaccgtagga
NM_080729	IL17e	gagtgggggtctcagctaca	gatggggctctgtaacctttc
NM_145856	IL17f	acttcagaggcgaaggcata	tgactgtgcatgttggattt
NM_008359	IL17ra	tttcatctttccagcctcct	ggagcccagttgtctgtgag
NM_134437	IL17rd	cagaagcgttggtagcagaa	ccactgagacagcaccagaa

Q-PCRs were run in triplicate and the values were transferred into copy numbers of DNA using a standard curve of genomic DNA with known copy numbers. The resulting transcription values for each gene were also normalized for primer pair amplification efficiency using the Q-PCR values obtained with Input DNA (unprecipitated genomic DNA). Results are presented as Transcription Events Detected Per 1000 Cells for each gene tested.

### Statistical Analysis

#### Morphometric analysis

We did not assume equal variance because the variance of PMN population is unknown; *p *values were calculated using a Welch's test (GraphPad Prism software version 4.0; GraphPad Software, Inc. San Diego, CA). A nominal *p *value less than 0.05 was considered statistically significant.

#### Q-PCR

The difference between two mean values was analyzed with an unpaired Student's *t*-test (GraphPad Prism software version 4.0; GraphPad Software, Inc. San Diego, CA). A nominal *p *value less than 0.05 was considered statistically significant.

#### Urinary Cytokines

The small sample sizes at each time point precluded the use of standard analysis methods such as *t*-tests comparing baseline to time X. To gain statistical power, urinary cytokine response over time was parameterized through a "mixed model" [59] according to the following equation:

Cytokine Level = α * Time + β * Trx * Time

where α and β are beta coefficients, Trx is BCG vs LPS & TNF-α, and Time is in days. If the Trx*Time interaction was found to be significant a Trx*Time^2 ^interaction was tested. Only the p value from the highest order interaction was reported. A variance component covariance matrix was used to account for the correlation structure inherent in the longitudinal measurements of cytokines. A cytokine was considered to be statistically significant if the nominal interaction p value was less than 0.05. All mixed model analysis was performed in the SAS system v 9.1.3 (Cary, NC).
